# One Health risk challenges and preparedness regarding bovine tuberculosis at abattoirs in North-central Nigeria: Associated drivers and health belief

**DOI:** 10.1371/journal.pntd.0010729

**Published:** 2022-09-06

**Authors:** Ismail Ayoade Odetokun, Nma Bida Alhaji, Jibrin Aminu, Mohammad Kabir Lawan, Madinat Abimbola Abdulkareem, Ibraheem Ghali-Mohammed

**Affiliations:** 1 Department of Veterinary Public Health and Preventive Medicine, University of Ilorin, Ilorin, Nigeria; 2 Department of Veterinary Public Health and Preventive Medicine, University of Abuja, Abuja, Nigeria; 3 Department of Public Health and Epidemiology, Niger State Ministry of Fisheries and Animal Resources, Minna, Nigeria; 4 Department of Veterinary Public Health and Preventive Medicine, Ahmadu Bello University, Zaria, Nigeria; Faculty of Medicine and Health Sciences, Universiti Putra Malaysia, MALAYSIA

## Abstract

**Background:**

Bovine tuberculosis (bTB) is a serious public health and neglected zoonotic disease responsible for 147,000 human cases and 12,500 deaths annually. This study assessed knowledge, risk perceptions, and preventive practices regarding bTB among occupationally exposed abattoir workers and drivers for transmission in slaughterhouses.

**Methods:**

Using a pre-tested questionnaire, we surveyed a cross-section of workers in five main abattoirs in North-central Nigeria between 2018 and 2019. Data were analysed using descriptive statistics and univariable/multivariable logistic regression analyses at a 95% confidence level.

**Results:**

All recruited respondents (n = 422: 77.7% meat processors and 22.3% meat and sanitary inspectors) participated and 10.4% had no formal education. About 44.0% and 27.0% of workers knew about bTB occurrence at the abattoirs and its transmission to humans, respectively. Less than one-third use personal protective equipment (PPE) during meat handling, only a few workers correctly practised routine handwashing, and 21.8% sterilized meat handling tools. A few participants (6.4%) had BCG vaccination against tuberculosis. Demographic characteristics (age, gender, occupation, and formal education) significantly influenced the perception and practices about bTB. A few workers perceived raw meat and milk, direct contact with infected carcasses, organs and contaminated fomites, contaminated environment through infected blood, dirty slaughtering floor, and aerosols of contaminated faeces as high-risk bTB transmission routes. Perceived drivers that influenced bTB transmission at abattoirs include unhygienic meat processing (OR = 5.4, 95%CI = 3.1–9.4, p < 0.001) and non-enforcement of abattoir standard operating systems (OR = 10.4, 95%CI = 6.0–18.5, p = 0.001).

**Conclusion:**

The workers have low knowledge levels, perceptions, and practices toward bTB emergence. These demand the workers’ education on hygienic meat handling to mitigate the menace of the disease. Surveillance and preventive preparedness considering the identified drivers through the ’One Health’ approach are recommended.

## Author summary

Bovine tuberculosis (bTB) is a neglected zoonotic disease of public health importance and responsible for high morbidity and mortality among humans in Africa. This study assessed knowledge, risk perceptions, and preventive practices regarding bTB among occupationally exposed abattoir workers and drivers for transmission in slaughterhouses. We found low knowledge levels and preventive measures of bTB among abattoir workers. Important drivers of bTB in the study area were also identified. This study contributed to the epidemiological findings on bTB, which will be helpful as guidelines for developing and implementing preventive measures. Institution of awareness programs and training abattoir workers will improve their knowledge of bTB and other zoonotic diseases.

## Introduction

Bovine tuberculosis (bTB) is a neglected zoonotic disease of cattle, wildlife, and humans, caused by *Mycobacterium bovis* of the *Mycobacterium tuberculosis* complex (MTC). The MTC comprises *M*. *tuberculosis*, the primary causative agent of human tuberculosis, and *M*. *canettii*, *M*. *africanum*, *M*. *pinnipedii*, *M*. *microti*, and *M*. *caprae* [1,2]. bTB is endemic in Nigeria and widespread in Africa [3–5]. *M*. *tuberculosis* and *M*. *africanum* have been isolated from cattle in Nigeria [6]. The disease is one of the economically important zoonoses worldwide, with substantial public health implications, high eradication costs, and restricted animal trade, especially in developing countries [7–9].

Tuberculosis (TB) is one of the leading infectious diseases responsible for human mortalities [10,11]. Most human TB cases are non-zoonotic, unlike those caused by most members of the MTC. Approximately, 10% of TB cases in Africa are zoonotic [12,13]. The WHO categorized zoonotic tuberculosis among seven neglected zoonotic diseases [14]. Globally, bTB caused an estimated 147,000 new human cases in 2015 and 12,500 deaths, with the highest incidence in Africa [15]. The WHO estimated that 10.4 million people had active TB cases and 1.7 million deaths worldwide in 2017 [16], which is an underestimation since many parts of the world do not have active TB or zoonotic TB surveillance. Currently, TB is underestimated due to the COVID-19 pandemic experienced since late 2019.

In developing countries, *M*. *bovis* infection is a significant challenge among human populations because humans and animals share a similar microenvironment. Approximately 8.5–9.2 million TB morbidities were documented, with 1.2–1.5 million mortalities in 2010 [17]. In Africa, it is projected that about 5–7% of all human TB cases are attributable to *M*. *bovis* [18]. Furthermore, 57.0% and 26.0% of the disease burden are disproportionately in Asia and Africa, respectively [17]. Consumption of raw milk and meat and inhaling contaminated aerosols from diseased cattle can lead to human infection. [19].

In Nigeria, bTB is detected mainly by meat inspection in slaughterhouses and less rarely by bacteriological techniques [20]. Herders, slaughterhouse workers, and other livestock and animal product handlers are at significant risk of *M*. *bovis* infection [5,20,21]. A 10% TB prevalence was recorded in a cross-section of sputum sampled from Nigerian livestock traders, with *M*. *bovis* strains detected in two of the seven TB cases from these occupational exposure individuals [22].

Information on the One Health risks and preventive practices regarding bTB among occupationally exposed workers in meat handling places is scarce. Understanding the threat of bTB infections is crucial, particularly in poor resource countries experiencing a high disease burden. These are also required for designing control programmes toward achieving the World Health Organization’s ‘END-TB’ agenda of curbing human TB as a public health concern by 2035. However, One Health risk and disease mitigation strategies can be verified using the Health Belief Model’s (HBM) "Perceived Vulnerability" construct, which asserts that people choose healthier behaviours when they sense personal risk or susceptibility. The stronger the risk perception of an illness, the more likely people are to participate in behaviours that reduce the disease’s risk [23,24].

The study objectives were to assess bTB risk at the human-animal-environment interface and preventive practices towards bTB among abattoir workers, risk routes for spread, and identify perceived drivers of bTB occurrence among the occupationally exposed meat handlers. We hypothesized that the demographic characteristics of the workers could not influence their perceptions of the risks of the disease. Outcomes of this study are expected to contribute to the surveillance and control information on the WHO’s ‘END-TB’ plan of eliminating all forms of human TB by 2035.

## Materials and methods

### Ethics statement

Participation in the survey was voluntary, and respondents were allowed to exit the study without prejudice. The respondents provided informed consent orally before the commencement of the interview. The confidentiality of the participants’ information was strictly maintained. No incentives were provided for participation. The Niger State Ministry of Livestock and Fisheries Research Ethics Committee approved the study (Ref: MLF/NGS/728).

### Study area

**Fig 1:**
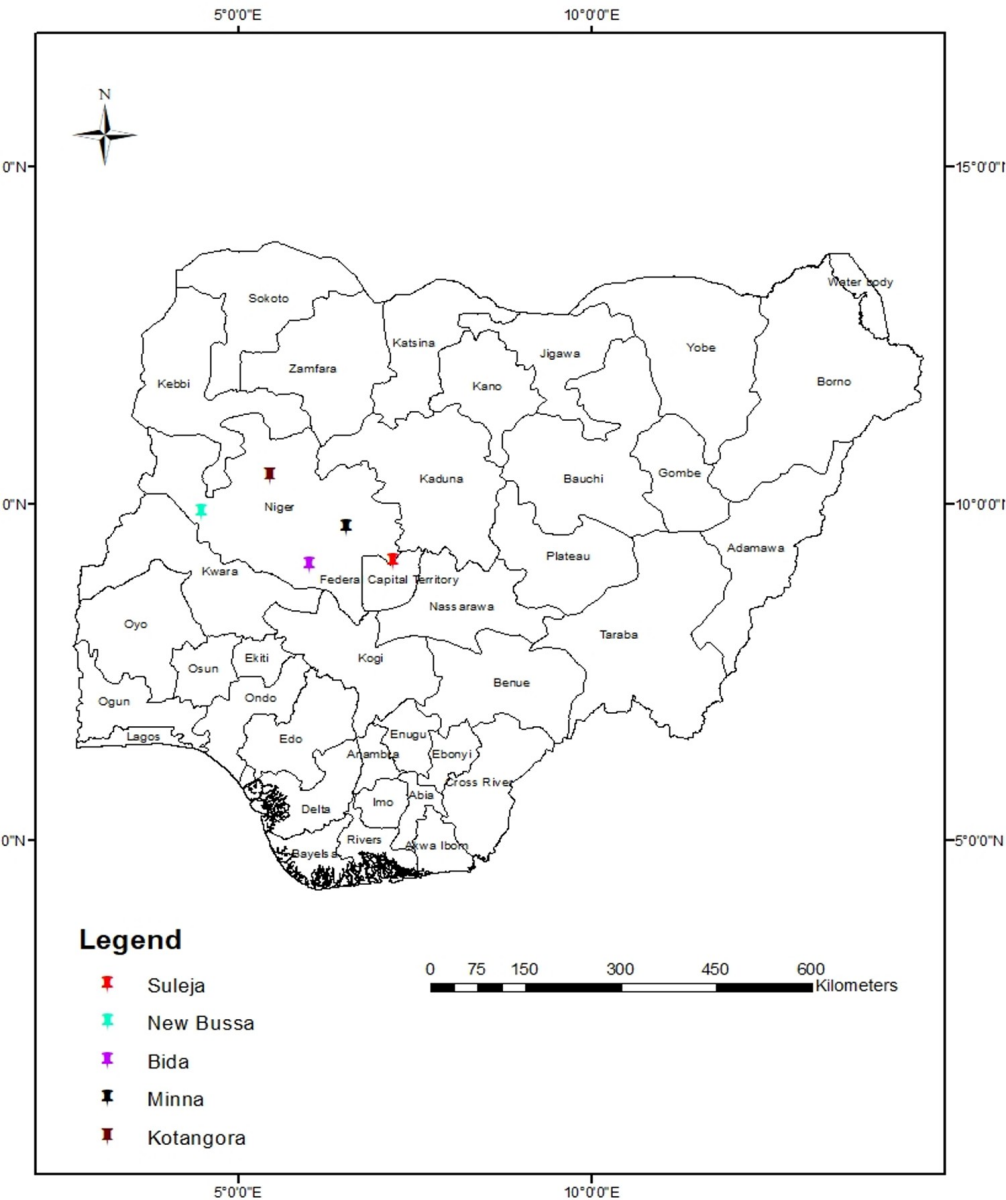
Map of the studied abattoirs in North central Nigeria. The map was created in ArcGIS-ArcMap version 10.3 (ESRI Co., Redlands, California, USA) using geocodes taken by Garmin eTrex 10. The base layer of the map used to generate the this figure was downloaded from https://esri.maps.arcgis.com/home/item.html?id=1563807f7a184e33826761281e2b31f3.

The research was conducted at five municipal randomly selected abattoirs in North-central Nigeria: Bida, Kontagora, Minna, New-Bussa, and Suleja (Fig 1). The abattoirs were selected using balloting. These abattoirs service high populations of meat consumers in these cities. The early dry season (October–December), late dry season (January–March), early rainy season (April–June), and the late rainy season (July–September) are the four distinct seasons in the study area. The average yearly rainfall is 1600 mm. It has an average low and high temperature of 27 and 39°C, respectively. As of the last population census in 2006, the study area has a human population of 4.94 million people [25].

### Study design, sample size, and sampling procedure

A questionnaire-based cross-sectional study was conducted on randomly selected workers in five municipal abattoirs in North-central Nigeria between January and December 2018. The workers were aged 20 years and above and available at the study sites during the survey. They were made up of meat processors (butchers and meat traders) and meat and sanitary inspectors. Workers under 20 years old, with a few years of experience on the job, and those with limited contact with slaughter animals were excluded from the study.

The sample size was calculated using the simple random formula, n = Z^2^ p (1—p)/d^2^ [26]. In this formula, n is the calculated sample size; Z^2^ is the standard deviation, 1.96; p is the expected response from the target populations, 50%; and d is the desired absolute precision, 5%. The open source epidemiologic statistics for public health (OpenEpi) version 2.3.1 software [27] was used to calculate the sample size resulting in 384 participants. We included a 10% contingency to make up for a possible incomplete questionnaire, and a sample size of 422 was obtained. Then, survey respondents were chosen using a simple random sample procedure to ensure that both men and women were included.

### Questionnaire development, structure, and data collection

A structured hard-copy questionnaire (S1 Text) was utilized to gather abattoir workers’ responses. A four-part questionnaire was developed. Part I recorded a total of demographic characteristics; Part II documented respondents’ knowledge about bTB; Part III was made up of perceptions on risk routes for transmission of bTB at abattoirs; Part IV contained questions on preventive practices, and Part V included perceived risk factors influencing bTB occurrence at abattoirs. The questionnaire was developed in English and then translated into *the Hausa* language for respondents without formal education. The survey tool was pre-tested with 30 respondents in a slaughterhouse, demonstrating similar features to the surveyed abattoirs. The slaughterhouse was consequently excluded from the main survey. A final adjustment was made to the survey tool before data collection. The questionnaire administration was interviewer-administered. Only English and *Hausa* local language speaking questionnaire trained administrators were involved in data collection.

### Data management and statistical analysis

Data obtained from the completed questionnaire in hard copies were entered into Microsoft Excel 2019 (Microsoft Corp. Redmond WA, USA) and coded before being subjected to statistical analysis. We summarized the data collected using descriptive statistics. A numeric scoring system was used to identify the knowledge levels of the abattoir workers. The outcomes were computed as binary responses of ‘No’ for incorrect answers and were scored ‘0’ and ‘Yes’ for correct responses and scored ‘1’ in which the total points were expressed as 100%. Univariable analyses (the Chi-square/ Fisher’s exact tests) were performed to determine a significant relationship between risk perceptions and transmission pathways, demographic variables and risk perception, and drivers of bTB transmission among abattoir workers. The risk perception on bTB transmission pathways was further categorized as low (<35%), moderate (35–65%), and high risk (>65%) and as binary–satisfactory (>50%) and poor (≤50%) to determine association with demographic variables. The greater the score, the greater the perception-preventive behaviour levels of the abattoir workers based on the HBM. The impacts of the independent factors (demographic variables and abattoir workers) on risk perception and drivers of bTB transmission (outcome variables) in the abattoir were then investigated using multiple logistic regression analysis. Variables included in the model were those with p-values less than 0.05 at the level of the univariable analysis. The least significant variable was excluded at each round of backward stepwise regression until the model comprised only those significant components at the confidence interval of 95%. Statistical analyses were performed using EpiInfo version 3.5.3 (CDC, Atlanta, USA), with statistical significance defined as a p-value of less than 0.05. The model’s quality of fit was assessed using the Hosmer-Lemeshow test.

## Results

### Demographic features of respondents

**Fig 2:**
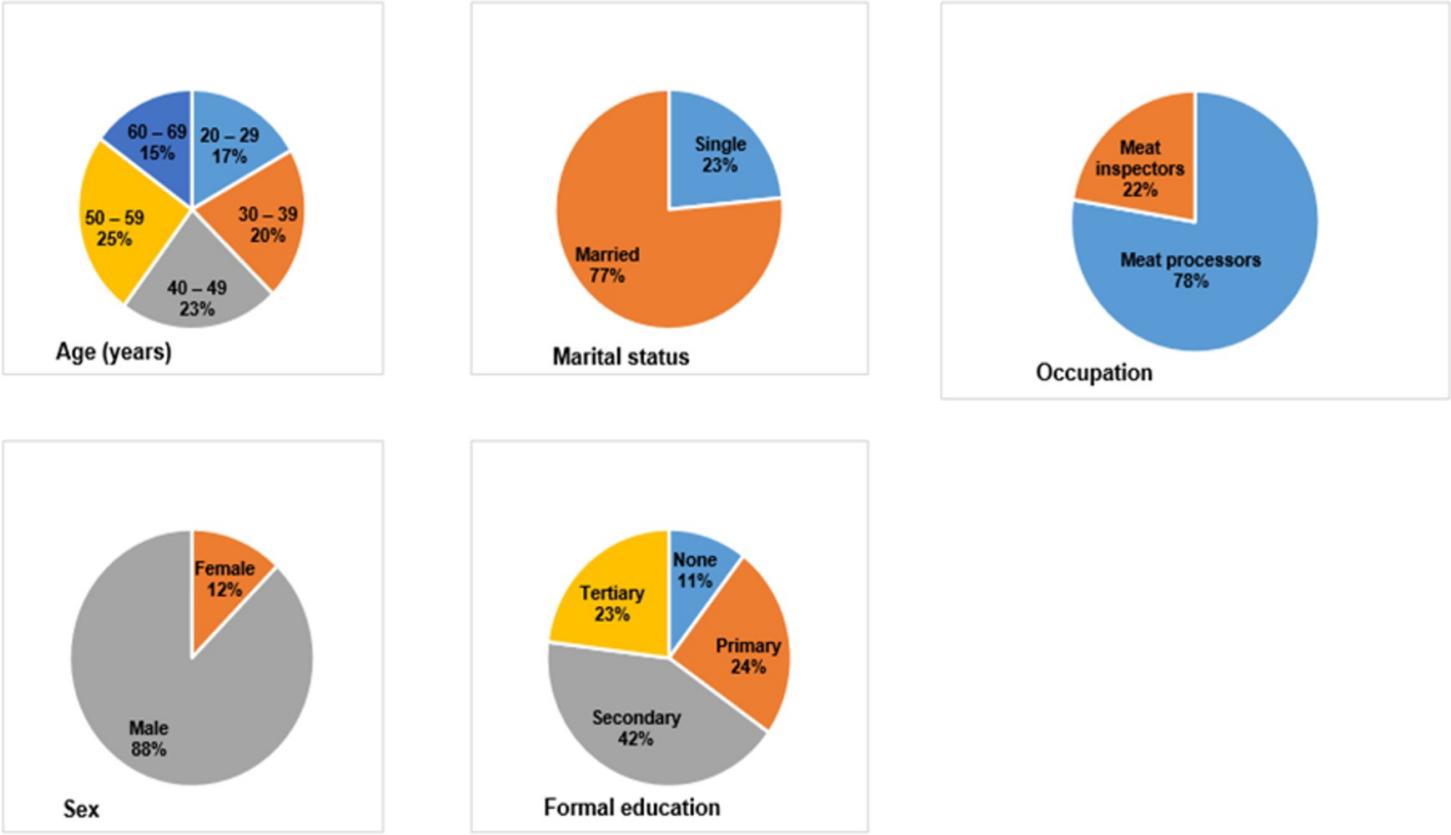
Demographic characteristics of abattoir workers at the North-central abattoirs in Nigeria.

A total of 422 abattoir workers participated in this study. Fig 2 presents the demographic features of the surveyed workers. Most participants were males (87.7%) and married (76.5%). The mean age of the participants is 50.5±15.5 SD years. Only 10.4% (44/422) of the respondents had no formal education, 24.4% (103/422) had primary education, 42.2% had secondary education, and 23.0% (97/422) of them possessed tertiary education. The majority of the surveyed workers were meat processors (77.7%).

### Respondents’ knowledge about bovine tuberculosis

Table 1 includes the responses on knowledge of bTB among the abattoir workers. Less than half of the workers knew about bTB as a disease, reported that bTB affects cattle only, and knew bTB to be zoonotic. Respondents also reported low awareness of the clinical signs of bTB in cattle and its capability to spread in abattoirs. Less than one-fifth of the respondents knew about the transmission routes to humans at abattoirs, likewise the clinical symptoms of bTB in humans.

**Table 1:** Abattoir workers’ knowledge about bovine tuberculosis in North-central Nigeria.

Variable	Yes n (%)	95% Confidence interval
Aware of bovine tuberculosis occurrence at the abattoir	184 (43.6)	38.9–48.4
Bovine tuberculosis affects only cattle	162 (38.4)	33.8–43.1
Know clinical signs of zoonotic tuberculosis in cattle	157 (37.2)	32.7–41.9
Bovine tuberculosis is contagious at abattoirs	118 (28.0)	23.8–32.4
Bovine tuberculosis is transmissible from animals to humans	113 (26.8)	22.7–31.2
Know routes of the disease spread to humans at abattoirs	79 (18.7)	15.2–22.7
Know clinical symptoms of bovine tuberculosis in humans	63 (14.9)	11.8–18.6

### Preventive practices by abattoir workers against bovine tuberculosis risks at abattoirs

The preventive measures practised by abattoir workers against bTB risks at abattoirs in Nigeria are presented in Table 2. Only 35% of workers practised routine hand washing with water and soap after meat handling. About 22% of them practised routine sterilization of meat handling tools. However, about 70% of workers adequately cook meat before consumption, while only 10% practised pasteurizing raw milk before consumption. Less than one-third practised the use of personal protective equipment during meat handling.

**Table 2:** Preventive measures practised by abattoir workers against bovine tuberculosis risks at abattoirs in Nigeria.

Practice	Yes n (%)	95% Confidence interval
Routine hand washing with water and soap after meat handling	148 (35.1)	30.6, 39.7
Routine disinfection of hands with sanitizers	102 (24.2)	20.3, 28.4
Routine washing and disinfection of abattoir floors	133 (31.5)	27.2, 36.1
Routine sterilization of meat handling tools	92 (21.8)	18.1, 25.9
Routine washing and disinfection of equipment	69 (16.4)	13.1, 20.1
Pasteurization of raw milk before consumption	41 (9.7)	7.2, 12.8
Adequate cooking of meat before consumption	297 (70.4)	65.9, 74.6
Isolation of suspected infected animals from healthy ones at lairage	58 (13.4)	10.7, 17.3
Use of personal protective equipment	121 (28.7)	24.5, 33.1
BCG vaccination against tuberculosis	27 (6.4)	4.4, 9.1

BCG: Bacille Calmette-Guérin

### Relationship of risk perceptions on the transmission pathways for bovine tuberculosis

Table 3 shows the risk perceptions of bovine tuberculosis transmission pathways at abattoirs in North-central Nigeria. Using HBM, very few workers significantly (p<0.05) perceived consumption of raw meat (8.5%) and raw offal (4.0%); the contaminated environment through aerosols of infected blood (6.6%), dirty slaughtering floor (10.0%), and aerosols of contaminated faeces (6.9%) to be a high risk of bTB.

**Table 3:** Risk perceptions on the transmission pathways for bovine tuberculosis at abattoirs in North-central Nigeria.

Pathways	Low risk n (%)	Moderate Risk n (%)	High risk n (%)	Chi-square	p-value
Consumption				37.9	<0.001
Raw meat	260 (61.6)	126 (29.9)	36 (8.5)		
Raw offal	194 (46.0)	211 (50.0)	17 (4.0)		
Direct contact with				33.7	0.001
Infected carcasses and organs	215 (50.9)	172 (40.8)	35 (8.3)		
Contaminated fomites	184 (43.6)	142 (36.6)	96 (22.8)		
Environment				46.8	<0.001
Aerosols of infected blood	189 (44.8)	205 (48.6)	28 (6.6)		
Unhygienic slaughtering floor	216 (50.6)	167 (39.4)	39 (10.0)		
Aerosols of contaminated faeces	279 (66.1)	114 (27.0)	29 (6.9)		

Note: Low risk (< 35%); Moderate risk (35–65%); High risk (> 65%); statistically significant at p < 0.05

### Demographic characteristics associated with risk perceptions about bovine tuberculosis

Abattoir workers’ demographic characteristics of age, gender, occupation, and formal education significantly influenced risk perception about bTB (Table 4). Older respondents were significantly more likely to have a satisfactory perception of bTB. Male workers were 2.6 times (95% CI: 1.43–4.81, p = 0.001) more likely to possess a satisfactory perception of bTB than female abattoir workers, similarly observed for meat and sanitary inspectors over meat processors. Furthermore, respondents with a higher level of formal education, such as tertiary education, were approximately 6.5 times (95% CI: 2.96, 14.32, p = 0.001) more likely to have a satisfactory perception of bTB than workers without formal education.

**Table 4:** Demographic characteristics associated with risk perceptions about bovine tuberculosis at abattoirs in North-central Nigeria.

Variable	Categories	Poor perception n (row %)	Satisfactory perception n (row %)	Chi-square test			Multivariate test		
				χ^2^	df	p-value	Odds ratio	95% CI	p-value
Age (y)	20–29	46 (64.8)	25 (35.2)	36.1	4	<0.001	1.0	-	-
	30–39	53 (60.9)	34 (39.1)				1.2	0.6, 2.3	0.623
	40–49	34 (44.2)	40 (55.8)				2.2	1.1, 4.2	0.020
	50–59	33 (31.4)	72 (68.6)				4.0	2.1, 7.6	0.001
	60–69	17 (27.0)	46 (73.0)				4.9	2.4, 10.4	0.001
Gender	Female	34 (65.4)	18 (34.6)	10.2	1	0.001	1.0	-	-
	Male	155 (40.9)	215 (59.1)				2.6	1.4, 4.8	0.001
Occupation	Meat processors	218 (66.2)	110 (33.8)	16.1	1	<0.001	1.0	-	-
	Meat and sanitary inspectors	41 (43.6)	53 (56.4)				2.6	1.61, 4.1	0.001
Formal education	None	31 (70.5)	13 (29.5)	28.1	3	<0.001	1.0	-	-
	Primary	56 (54.4)	47 (45.6)				2.0	0.9, 4.3	0.070
	Secondary	87 (48.9)	91 (51.1)				2.5	1.2, 5.1	0.010
	Tertiary	26 (26.8)	71 (73.2)				6.5	2.9, 14.3	0.001

CI–Confidence interval; Statistically significant at p<0.05; χ^2^ –chi-square test; df–degree of freedom; y—years

### Perceived drivers of bovine tuberculosis transmission at abattoirs in North-central Nigeria

As reported by abattoir workers at the slaughterhouses, several perceived drivers that significantly influenced bTB emergence and transmission were identified (Table 5). Meat inspectors were at least 3.5 times more likely to agree that unhygienic meat processing (95% CI: 3.1–9.4, p < 0.001), non-use of personal protective equipment (95% CI: 2.5–6.6, p < 0.001), unsanitary environmental conditions (95% CI: 2.9–8.5, p < 0.001), inadequate or absence of antemortem examinations (95% CI: 3.8–11.5, p < 0.001), non-enforcement of abattoir standard operating system (95% CI: 6.0–18.5, p = 0.001), socio-economic status (poverty) of operators (95% CI: 3.6–10.8, p < 0.001), and lack of training or re-training on proper meat handling (95% CI: 2.2–5.6, p = 0.001) can be a factor influencing the transmission of bTB in the abattoirs in North-central Nigeria.

**Table 5:** Factors influencing bovine tuberculosis transmission among abattoir workers in North-central Nigeria.

Factor	Disagree (%)	Agree (%)	Chi-square test			Multivariate test		
			χ^2^	df	p-value	Odds ratio	95% CI	p-value
Unhygienic meat processing								
Meat processors	184 (56.1)	144 (43.9)	39.9	1	<0.001	1.0	-	-
Inspectors	18 (19.1)	76 (80.9)				5.4	3.1, 9.4	<0.001
Non-use of personal protective equipment								
Meat processors	207 (63.1)	121 (36.9)	32.9	1	<0.001	1.0	-	-
Inspectors	28 (29.8)	66 (70.2)				4.0	2.5, 6.6	<0.001
Unsanitary environmental condition								
Meat processors	193 (58.8)	135 (41.2)	38.9	1	<0.001	1.0	-	-
Inspectors	21 (22.3)	73 (77.7)				4.9	2.9, 8.5	<0.001
Inadequate or absence of antemortem examinations								
Meat processors	200 (61.0)	128 (39.0)	51.2	1	<0.001	1.0	-	-
Inspectors	18 (19.1)	76 (80.9)				6.6	3.8, 11.5	<0.001
Non-enforcement of abattoir standard operating system								
Meat processors	238 (72.6)	90 (27.4)	84.1	1	<0.001	1.0	-	-
Inspectors	19 (20.2)	75 (79.8)				10.4	6.0, 18.5	0.001
Socio-economic status (poverty level) of operators								
Meat processors	206 (62.8)	122 (37.2)	50.7	1	<0.001	1.0	-	-
Inspectors	20 (21.3)	74 (78.7)				6.3	3.6, 10.8	<0.001
Inadequate training on proper meat handling								
Meat processors	239 (72.9)	89 (27.1)	28	1	<0.001	1.0	-	-
Inspectors	41 (43.6)	53 (56.4)				3.5	2.2, 5.6	0.001

CI–Confidence interval; Statistically significant at p<0.05; χ^2^ –chi-square test; df–degree of freedom

## Discussion

To achieve the World Health Organization’s ‘END-TB 2035’ goal using the One Health approach, adequate knowledge, perceptions, and practices towards bTB among the occupationally exposed individuals are crucial. This is particularly important as bTB contributes to human TB cases. Thus, an approach at the human, animal, and environmental nexus must be effectively adopted as launched by the tripartite WHO/FAO/OIE [16] roadmap for zoonotic tuberculosis.

This study focuses on testing the knowledge and perception of abattoir workers in Nigeria on bTB and their level of effective practices adopted to prevent this disease. This study elucidated that about 44% of the surveyed workers knew bTB as a disease. A study in Zamfara, Nigeria [28] showed bTB knowledge level as 46.30% among the surveyed abattoir workers. Likewise, only 15.0% of (abattoir workers and herders) and 32.55% (of cattle owners) were knowledgeable about bTB in a study in Karachi, Pakistan [29] and eastern Ethiopia [30], respectively. The discrepancy in knowledge level might be due to the different study locations and population settings.

The finding that only 26.8% of the respondents were aware of the zoonotic potential of bTB is disturbing, considering that over 50.0% of them lacked adequate knowledge of bTB despite their high exposure to this disease. This is slightly lower than the knowledge level (30.5%) of abattoir workers in Zamfara, Nigeria [28], and a bit higher than the 23.3% among cattle owners in Ethiopia [30]. Agada et al. [31] reported that 50% of livestock workers in Lafia town, Nigeria, have good knowledge about bTB. A study conducted in Ethiopia recorded a higher (95.3%) knowledge level of transmissibility of bTB from animals to humans [32]. Good knowledge and awareness of bTB are crucial for controlling the disease spread from animals to humans.

On bTB preventive practices, our findings showed that the surveyed abattoir workers do not practice optimum preventive practices, further exacerbating their risk of contracting the disease. Although 70.0% reported adequate meat cooking before consumption, all other practices were below average. Aduloju et al. [33] reported that only 30.4% of abattoir workers in Ibadan, Nigeria were aware of bTB transmission to humans through consuming infected unpasteurized milk. Poor disease prevention strategies are commonly observed among abattoir workers and milk processors in Nigeria [34, 35].

The current low adoption of preventive practices in this study is of great public health concern. This can be due to a lack of awareness of the disease by the workers and a lack of training PPE usage and other preventive measures. These findings are similar to low preventive measures adopted by some abattoir workers as described by Ismaila et al. [28], Adesokan et al. [36], Odetokun et al. [37], and Memon et al. [29]. This calls for a One Health approach to controlling TB, and all stakeholders at different levels need to be educated and fully equipped on the preventive measures to be taken.

We found that few workers significantly perceived consuming raw meat as high-risk behaviour. Earlier studies in Nigeria and central Ethiopia showed that most abattoir workers in Nigeria do not perceive the consumption of raw or undercooked meat as a means of bTB transmission to humans [28,32,38]. Similar to our findings, consumption of raw offals, contact with infected carcasses and organs from processed animals, contaminated environment and fomites, and poor hygienic conditions of the slaughterhouses have been identified as high bTB risks in other studies [29,32,33,38]. This further explains the knowledge gap of abattoir workers on preventive practices. Hence, abattoir workers need to be educated on the zoonotic pathways of bTB and other diseases.

Age, gender, occupation, and formal education significantly influenced risk perception about bTB. The demographic characteristics associated with risk perceptions about bovine tuberculosis showed satisfactory risk perception levels among respondents with higher age categories, especially among the males, meat and sanitary inspectors, and those with higher educational levels. This is expected in African countries as males are always at the forefront of tasking events due to social masculinity and have more active years than females. Meat and sanitary inspectors significantly had higher knowledge than meat processors. Abattoir workers with secondary and tertiary education significantly had more knowledge than others. Education level has been shown to impact knowledge of risk perception, and workers with post-primary education were more knowledgeable than those without formal education [29,35].

The reported perceived drivers influencing bTB emergence and transmission at abattoirs are comparable to other findings. Fekadu et al. [32] reported that provision of free PPE, enforcement of standard operating system, and compensation of owners of condemned meats were found to help protect the public against bTB. The use of PPE influenced good practice among slaughterhouse workers [34,39]. Comparably, abattoir workers knowledgeable about improper waste disposal impacts and the effects of improper operations on public and environmental health were more likely to demonstrate acceptable preventive practices in slaughterhouses [39]. Our results underscore the need for focused interventions on education and institution of economic support programmes for abattoir workers.

African countries, including Nigeria, are expected to prioritize One Health focusing mainly on challenges affecting human and animal health and the environment [40]. Our observations from this study have supported utilizing the One Health approach to prevent bTB outbreaks among animals and humans while improving food safety and security. One Health focuses on a multi-sectorial and multi-disciplinary approach to solving zoonotic threats [41–43]. Relevant governmental agencies/officials, researchers, medical doctors, including veterinarians, and other stakeholders across all levels in Nigeria should be involved in controlling the threats emanating from bTB, as previously emphasized [41]. Particularly, it should be directed at the drivers of bTB highlighted in the study using the right approach [44] to achieve the expected benefits [45].

Although this study gathered epidemiological data from abattoir workers in five municipal abattoirs in North-central Nigeria through a structured questionnaire, it has limitations. The main limitation concerns the use of a cross-sectional design which does not show a causal relationship among the abattoirs. However, using confidence intervals in our analyses has taken care of the imperfections possibly associated with sampling in the abattoirs. The study was conducted in one of the country’s six geopolitical zones. However, the findings from this study are generalizable to other abattoirs in Nigeria, as the structural conditions and enforcement regulations are the same across the country. This study also recruited more males than females reflecting the workforce distribution by gender across other slaughterhouses in Nigeria [34,37,46–48].

## Conclusion

The abattoir workers lack adequate knowledge about bTB as a disease and its zoonotic transmissibility to humans. They show a poor adoption level of preventive practices. A high knowledge level about bTB is expected from these workers saddled with the responsibility of ensuring food safety and adopting good practices to prevent harbouring and transmitting diseases to the general public. Thereby, different measures such as training on proper meat handling; enlightenment programs on bTB and other diseases; awareness of various zoonotic diseases pathways; occupational hazards and safety protocols to uphold as well as preventive practices have to be put in place to curb the menace that might arise from these challenges. Furthermore, surveillance and preventive preparedness that consider the perceived drivers through the ’One Health’ approach are urgently needed to contribute to the World Health Organization’s ’END-TB’ goal of eliminating all forms of human tuberculosis as a public health problem by 2035.

## Supporting information

S1 Text Questionnaire used in the survey. (DOCX)
