# Supplementary material for: One Health risk challenges and preparedness regarding bovine tuberculosis at abattoirs in North-central Nigeria: Associated drivers and health belief
Source: PLoS Negl Trop Dis. 2022 Sep 6;16(9):e0010729. doi: 10.1371/journal.pntd.0010729 (PMC9481158; doi:10.1371/journal.pntd.0010729)
Supplement: S1 Text — (DOCX) [file pntd.0010729.s001.docx]

**QUESTIONNAIRE**

We are conducting a survey on knowledge, zoonotic risk perceptions and preventive preparedness on bovine tuberculosis in slaughtered cattle at abattoirs in North-central Nigeria. You have been identified to participate and all information given will be kept strictly confidential. ***PLEASE circle one or more*** *appropriately and* ***write*** *where necessary****.***

Name of abattoir……………………… Location…………………… Date of interview…………………..

1. **Socio-demographic Information**

A1. Name of person interviewed…………………………………..

A2. Age (in years)…………………

A3. Sex: a. Male ( ) b. Female ( )

A4. Marital Status: a. Single ( ) b. Married ( )

A5. What is your role (occupation) in the abattoir?

1. Meat inspector/Animal health official ( ) b. Butcher ( ) c. Sanitary officer ( ) d. Meat seller ( )

A6. Highest formal educational level:

1. None ( ) b. Primary ( ) c. Secondary ( ) d. Tertiary ( )
2. **Knowledge about Bovine Tuberculosis**

B1. Have you ever heard about bovine tuberculosis? No ( ) Yes ( )

B2. If Yes, from what source? a. Radio ( ) b. Friends ( ) c. Relations ( ) d. Veterinary/health authorities ( ) e. Others (please specify)­­­­­­­­­­­­­­­­­­­­­---------------------------

B3. Have you ever seen live cattle with tuberculosis? Yes ( ) No ( )

B4. If Yes, what are the clinical manifestations observed in animals?

1. Fluctuating fever ( ) b. Intermittent hacking cough ( ) c. Weight loss ( ) d. Loss of appetite ( ) e. Diarrhea ( ) f. All of the above ( )

B5. Have you ever seen slaughtered cattle with tuberculosis lesions at post-mortem? Yes ( ) No ( )

B6. If Yes, which organs are frequently affected? A. Lungs ( ) b. Liver ( ) c. Intestines ( ) d. Lymph nodes ( ) e. Others (please specify)…………………………………

B7. What is the trend of bovine tuberculosis occurrence in slaughtered cattle in this abattoir/market?

1. Decreasing ( ) b. Unchanged ( ) c. Increasing ( )

B8. Can bovine tuberculosis be transmitted from animals to humans (zoonosis)? Yes ( ) No ( ) Don’t know ( )

B9. If Yes, what are the clinical symptoms manifested in humans? a. Fever ( ) b. Night sweat ( ) c. Persistent cough ( ) d. Weight loss ( ) e. Abdominal pain ( )

B10. Is bovine tuberculosis routinely found (endemic) in environment? Yes ( ) No ( ) Don’t know ( )

B11. What are the factors that could predispose to zoonotic tuberculosis occurrence among meat handlers?

1. Inhalation of aerosol droplets from infected cattle ( )
2. Handling of infected tissues at post-mortem ( )
3. Handling of infected blood ( )
4. Contacts with infected bodily fluids ( )
5. Consumption of contaminated meat ( )
6. Handling contaminated milk ( )
7. Ignoring safety standards for hygiene at abattoir ( )
8. **Perceptions about zoonotic Risk of Bovine Tuberculosis at Slaughtered Houses**

C1. Consumption of raw meat: No risk ( ) Low risk ( ) Moderate risk ( ) High risk ( )

C2. Handling blood of infected animals: No risk ( ) Low risk ( ) Moderate risk ( ) High risk ( )

C3. Handling bodily fluid of infected animals: No risk ( ) Low risk ( ) Moderate risk ( ) High risk ( )

C4. Handling meat of infected animals: No risk ( ) Low risk ( ) Moderate risk ( ) High risk ( )

C5. Handling contaminated milk: No risk ( ) Low risk ( ) Moderate risk ( ) High risk ( )

C6. Consumption of contaminated milk: No risk ( ) Low risk ( ) Moderate risk ( ) High risk ( )

1. **Preventive Practices against Bovine Tuberculosis**

D1. Do you have training in meat hygiene? Yes ( ) No ( )

D2. Do you have training on environmental sanitation in meat handling premises? Yes ( ) No ( )

D3. Do you use protective coverings during meat handling or processing? Yes ( ) No ( )

D4. If Yes, what type(s)? a. Apron ( ) b. Face mast ( ) c. Hand glove ( ) d. Robber boots ( ) e. Use of eye goggle ( ) f. All of the above ( )

D5. What other preventive measures do you practice in this slaughterhouse?

a. Wash hands with soap after touching raw meat ( ) b. Clean cutting utensils and surfaces after meat operation ( ) c. Proper waste collection and disposal ( ) d. Frequent cleaning and disinfection of the meat operation environment ( ) e. All of the above ( ) f. None ( )

D6. Do unhygienic and unsanitary operations in this abattoir have effects on health? Yes ( ) No ( )

D7. If Yes, which health do they affect? a. Human health only ( ) b. Animal health only ( ) c. Environmental health only ( ) d. All of the above ( )

D8. How are the condemned organs and other waste products associated with bovine tuberculosis disposed off in this abattoir? a. Burn in ditch ( ) b. Disposed into drainages ( ) c. Incineration ( ) d. Buried in pits ( ) e. All of the above ( )

D9. What types of drainage systems exist in this abattoir? a. City central drainage ( ) b. Closed systems into farms ( ) c. Run-off into streams ( ) d. Into septic tank ( ) e. None ( )

D10. Do you use disinfectant during cleaning exercises in this abattoir? Yes ( ) No ( )

D11. If Yes, what type(s)? a. Izal ( ) b. Dettol ( ) c. Morigan ( ) d. Detergents ( ) e. Others (please specify)………………………………………………..

Thank you for the response

**Correspondent: GSM:** +234 8035950915;

**Email:** [nmabida62@gmail.com](mailto:nmabida62@gmail.com); odetokun.ia@unilorin.edu.ng
